# GENet: A Geometry-Enhanced Network for LiDAR Semantic Segmentation

**DOI:** 10.3390/s26051460

**Published:** 2026-02-26

**Authors:** Yuchen Wu, Hanbing Wei

**Affiliations:** School of Mechatronics and Vehicle Engineering, Chongqing Jiaotong University, Chongqing 400074, China; ryanwu@cqjtu.edu.cn

**Keywords:** LiDAR point cloud, semantic segmentation, range view, geometry attention, feature fusion

## Abstract

LiDAR has been widely applied in autonomous driving and mobile robotics. Recently, many studies focus on real-time point cloud segmentation, aiming to achieve higher accuracy while maintaining real-time inference speed. Current real-time methods mostly rely on 2D projection, which inevitably leads to spatial information loss. To address the limitations of 2D projection methods, we propose a Geometry-Enhanced Network called GENet that exploits spatial priors. The network employs an Atrous Separable Range Attention (ASRA) module to explicitly utilize spatial priors from range images, enabling geometry-aware feature aggregation with large receptive field at linear complexity. A Geometry-Context Modulation (GCM) mechanism is then used to calibrate semantic features, incorporating geometric priors while preserving the discriminative ability of original features across different categories. Experiments show that our method achieves efficient information fusion while maintaining real-time performance. Compared to existing methods, GENet requires fewer parameters and less computation, achieving a favorable balance between accuracy and efficiency.

## 1. Introduction

In recent years, with the rapid development of edge computing chips and the wide application of mobile robots in various scenarios, LiDAR has become a popular sensor choice due to its accuracy and robustness. Compared to other 3D sensors like RGB-D cameras and stereo cameras, LiDAR has natural advantages such as long sensing range and robustness to lighting conditions. Therefore, LiDAR-based environment perception, especially 3D point cloud semantic segmentation, has been a research focus in 3D computer vision.

LiDAR point clouds are direct samplings of real scenes, with characteristics like irregularity, sparsity, and non-uniformity, making them difficult for deep neural networks to learn. To handle this problem, there are several approaches. Point-based methods operate directly on raw point clouds, generally following the PointNet++ [1] paradigm to obtain local geometric shapes through hierarchical sampling and aggregation. Some of these methods achieve high segmentation accuracy, but the per-point processing and extensive 3D spatial searches cause high computational complexity, failing to meet real-time requirements. Voxel-based methods discretize point clouds into regular 3D grids and use existing 3D convolutions for feature learning. However, the computation and memory costs of 3D convolutions grow cubically with voxel resolution, making them unsuitable for resource-limited platforms. Projection-based methods project point clouds onto 2D planes (e.g., range view, bird’s-eye view) and use efficient 2D convolutional networks for processing, offering strong speed advantages. However, the spatial geometric information loss from projection leads to lower segmentation accuracy than the above two methods. In summary, how to balance accuracy and speed in point cloud semantic segmentation has become a hot research topic. Some studies propose multi-view fusion methods, incorporating the complementarity of different view representations into spatial prior information. For example, range views preserve neighborhood scanning structures while bird’s-eye views preserve spatial structures, which further improves the accuracy of multi-view methods.

**Figure 1:**
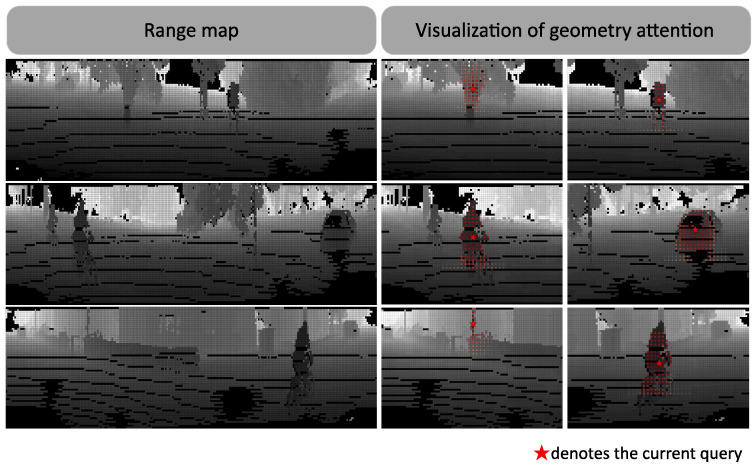
Illustration of geometry attention. The left shows the range image of the current frame, and the right shows the geometry attention responses at different query, where the “red star” denotes the current query. In the visualization map, red indicates the attention score of the current point, while gray represents the range value.

Although multi-view fusion methods have excellent segmentation accuracy, multi-branch networks significantly increase parameter count and model complexity compared to single-branch networks, reducing inference speed. Therefore, this paper considers a new perspective for introducing geometric prior information, enhancing point cloud semantic segmentation performance while maintaining computational efficiency. Unlike using separate neural network branches to encode prior information, we propose a learning-free geometry attention module that can be embedded into any backbone network. As shown in Figure 1, this module explicitly uses range images to guide feature aggregation and modulates original features by combining semantic and geometric context. Experiments show that our method achieves efficient information fusion while maintaining real-time performance. Compared to other methods, it requires fewer parameters and less computation, achieving a favorable balance between accuracy and real-time performance.

The main contributions of this paper are as follows:

- We introduce a learning-free geometry attention mechanism for point cloud semantic segmentation, enhancing network capability without additional learnable parameters.
- We design a Geometry-Enhanced (GE) Block that efficiently fuses 2D semantic features with 3D geometric cues. We develop two network architectures, GENet and GENet-Light, tailored for different efficiency requirements.
- We propose a novel range view interpolation strategy that efficiently searches for potential interpolation points and performs interpolation based on spatial distance.
- Comprehensive experiments on SemanticKITTI and SemanticPOSS demonstrate that our method achieves an optimal trade-off between accuracy and efficiency.

## 2. Related Work

**Point Cloud Semantic Segmentation:** Point cloud semantic segmentation is a fundamental task in computer vision, aiming to assign point-wise semantic labels. Point-based methods [2,3,4,5] follow the PointNet++  paradigm, directly processing raw point clouds by partitioning them into multiscale point sets through hierarchical sampling, then employing shared MLPs or transformer blocks to capture local geometric features. Due to the irregularity and sparsity of point clouds, many methods organize them into structured 2D representations, such as range views or bird’s-eye views, to leverage efficient 2D visual backbones. To restore 2D segmentation results to 3D space, RangeNet++ [6] proposes a KNN-based post-processing method, while FIDNet [7] employs an interpolation-based decoder with dilated convolutions and a nearest assignment strategy, improving efficiency while maintaining accuracy. CENet [8] utilizes a carefully designed training architecture with multi-scale auxiliary losses, achieving state-of-the-art results among projection-based methods.

Despite their efficiency, projection-based methods inherently suffer from spatial information loss, preventing them from matching the performance of point-based approaches. Some works attempt to concatenate additional geometric information to the input, such as normal maps [9], but with limited effectiveness. Therefore, how to effectively recover the geometric information lost during projection without incurring excessive computational overhead has become a key challenge for improving projection-based methods.

**Feature Fusion:** To address these challenges, researchers have extensively explored feature-level fusion strategies to exploit the complementarity of multi-modal or multi-view information. Recent works have investigated efficient fusion of information from different modalities, such as RGB, point clouds, optical flow, and depth maps, which provide additional contextual cues to enhance model understanding. In the RGB-D segmentation domain, numerous studies have demonstrated that feature-level fusion is an effective paradigm. CMX [10] builds a general multi-modal feature interaction framework using cross-attention; building upon this, AsymFormer [11] further optimizes computational resource allocation with an asymmetric dual-branch architecture for efficient segmentation. In point cloud segmentation, researchers have noted the complementary nature of different views and proposed multi-view feature fusion methods to compensate for spatial information compression caused by single-view projection. RPVNet [12] employs range, voxel, and point branches to extract features from different views, performing multi-level gated fusion across multiple stages. 2DPASS [13] additionally utilizes 2D images during training to generate semantic priors at multiple scales, and introduces texture and color priors into the 3D backbone through multi-scale multi-modal fusion distillation. Additionally, SqueezeSegV3 [14] explores incorporating more information within a single view, proposing a spatially adaptive convolution module that implicitly leverages spatial priors in a single-branch network.

Despite these advancements, several challenges persist: multi-view fusion methods achieve higher accuracy but the multi-branch architecture significantly increases computational overhead; single-view methods often sacrifice real-time performance when incorporating geometric information. Therefore, how to effectively fuse geometric priors while maintaining single-branch network efficiency remains an open problem worth exploring.

## 3. Materials and Methods

### 3.1. Problem Definitions

Recent studies have proposed projecting point clouds into spherical coordinates to obtain a compact 2D representation, addressing the inherent sparsity of LiDAR sampling in outdoor scenes. This approach enables the use of mature 2D visual backbones, making real-time 3D semantic segmentation feasible. For any point (x,y,z) in space, given the vertical and horizontal resolution (H,W) of the range view, the corresponding projected coordinates (u,v) can be computed as:

(1) $$\left(\begin{array}{c} u\\ v \end{array}\right)=\left(\begin{array}{c} \frac{1}{2}\left[1-\arctan\left(y,x\right)\pi^{-1}\right]W\\ \left[1-\left(\arcsin\left(zr^{-1}\right)+f_{\mathrm{up}}\right)f^{-1}\right]H \end{array}\right)$$

where,

(2) $$r=\sqrt{x^{2}+y^{2}+z^{2}}$$

denotes the distance from each point to the sensor, and

(3) $$f=|f_{\mathrm{up}}\left|+\right|f_{\mathrm{down}}|$$

denotes the vertical field of view of the LiDAR, with $f_{\mathrm{up}}$ and $f_{\mathrm{down}}$ representing the pitch angles. The result is a 5×H×W multi-channel 2D image, where each pixel contains 5 channels (x,y,z,r,i), representing the point coordinates, range, and reflectance intensity, respectively. Although this transformation converts point cloud segmentation into a 2D multi-channel image segmentation problem, addressing the unordered and sparse nature of point clouds, it compresses spatial information, preventing 2D convolutions from capturing local geometric structures and leading to erroneous feature aggregation. The goal of this work is to learn a geometry-aware feature enhancement transformation for a 2D point cloud feature map $X\in R^{C\times H\times W}$ and a range image $R\in R^{1\times H\times W}$:

(4) Y=F∗(X,R;Θ)

This transformation should satisfy two design principles: (1) points that are spatially proximate in 3D space should exhibit stronger feature interactions; (2) the semantic discriminability of the original features should be preserved.

### 3.2. Method Overview

**Figure 2:**
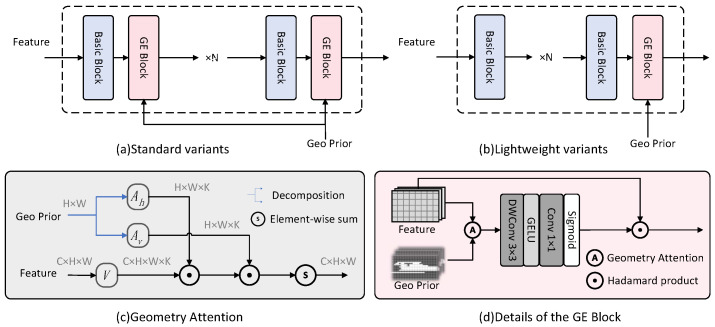
For each stage of the backbone: (**a**) GENet (standard version) inserts a GE Block after each feature extraction block for fine-grained geometry-semantic fusion. (**b**) GENet-Light (lightweight version) inserts a single GE Block only at the output of each stage for feature enhancement, enabling faster inference. (**c**) Detailed illustration of the proposed geometry attention module. (**d**) Detailed structure of the GE Block.

This paper proposes a geometry-aware feature enhancement module (GE Block) for range view point cloud semantic segmentation. The network architecture is illustrated in Figure 2. The module can be integrated with any 2D feature extraction backbone to enhance geometric awareness; we adopt ResNet [15] blocks for fair comparison with prior works. The module comprises two core components: (1) Geometry Attention Module (GAM), which combines atrous sampling with separable attention design, achieving geometry-aware feature aggregation with a large receptive field while maintaining linear computational complexity; (2) Geometry-Context Modulation (GCM), which transforms geometric context into channel-wise enhancement/suppression signals, incorporating geometric priors while preserving the discriminability of original features.

### 3.3. Geometry Attention Module

#### 3.3.1. Geometry Prior Generated from Range Image

Let *q* denote the query with coordinates $p_{q}$, and *k* denote other points within the window with coordinates $p_{k}$. The Euclidean distance between them is defined as

(5) $$d_{qk}={∥p_{q}-p_{k}∥}_{2}.$$

A Gaussian kernel is applied to map this distance to attention weights

(6) $$a_{qk}=\exp\left(-\frac{d_{qk}^{2}}{\tau }\right),$$

where τ is a learnable factor that controls the decay rate of attention with respect to distance, adapting to geometric characteristics at different scales. Through this geometry window attention mechanism, the model effectively captures spatial relationships of point clouds while maintaining computational efficiency.

#### 3.3.2. Atrous Separable Range Attention Module

Although geometry window attention effectively captures local geometric relationships, directly applying it on high-resolution feature maps incurs $O(H\cdot W\cdot K^{2})$ computational and memory overhead. Inspired by sparse attention research, we propose **Atrous Separable Range Attention (ASRA)**, which reduces complexity to O(H·W·2K) through axial decomposition, significantly improving efficiency. Specifically, standard K×K window attention requires each query to interact with $K^{2}$ keys; ASRA employs axial decomposition, first computing attention with *K* points along the horizontal axis, then with *K* points along the vertical axis. The cascaded operations cover the same K×K neighborhood, but the total number of interactions is only 2K. Meanwhile, atrous sampling is introduced to maintain a large receptive field without increasing the number of sampling points. Furthermore, different dilation rates can be set for horizontal and vertical directions to mitigate horizontal distortion in range views at different resolutions.

Specifically, given input features $F\in R^{C\times H\times W}$ and range image $R\in R^{1\times H\times W}$, the fixed-window attention is decomposed into horizontal and vertical attention. Each query point computes attention with *k* neighboring points along horizontal and vertical directions with strides $d_{h}$ and $d_{v}$ following (6). Thus, for each query point (i,j), the attention weight $A_{i,j}^{\left(k\right)}$ for its *k*-neighborhood is computed as:

(7) $$A_{i,j}^{\left(k\right)}=\exp\left(-\frac{{\left(r_{i,j}-r_{i,j+k\cdot d}\right)}^{2}}{\tau }\right)$$

where *K* is the number of sampling points, *d* is the dilation rate, and τ is the decay rate. Through atrous sampling, the effective receptive field is expanded to (K−1)·d+1. In our implementation, we set K=17, $d_{h}=d_{v}=2$, and τ is initialized to 1.0, achieving an effective receptive field of 33 pixels in both horizontal and vertical directions.

This yields the attention weight matrices for horizontal and vertical directions, denoted as $A_{h}$ and $A_{v}$, respectively. The geometry-prior aggregated feature maps $F_{h}$ and $F_{v}$ can then be computed as:

(8) $$F_{i,j}^{\left(h\right)}=\sum_{k=-\lfloor K/2\rfloor }^{\left\lfloor K/2\right\rfloor }A_{i,j+k\cdot d_{h}}^{\left(h\right)}\cdot F_{i,j+k\cdot d_{h}}$$

(9) $$F_{i,j}^{\left(v\right)}=\sum_{k=-\lfloor K/2\rfloor }^{\left\lfloor K/2\right\rfloor }A_{i+k\cdot d_{v},j}^{\left(v\right)}\cdot F_{i+k\cdot d_{v},j}$$

Then, through cascaded operations, the final aggregated feature map C is obtained. The entire module can be formulated as:

(10) $$\mathrm{ASRA}\left(F,R\right)=A_{v}\left(A_{h}\left(F,R;d_{h}\right),R;d_{v}\right)$$

The complete procedure of ASRA is summarized in Algorithm 1.
**Algorithm 1** Atrous Separable Range Attention (ASRA)1:**Input:** Feature map $F\in R^{C\times H\times W}$, Range image $R\in R^{1\times H\times W}$, Kernel size *K*, Dilation rates $d_{h}$, $d_{v}$, Learnable decay rate τ2:**Output:** Geometry-aware aggregated feature $F_{out}$3:// Horizontal Attention4:**for** each position (i,j) **do**5:    **for** k=−⌊K/2⌋ to ⌊K/2⌋ **do**6:        $d_{qk}\leftarrow \left|r_{i,j}-r_{i,j+k\cdot d_{h}}\right|$ // Range distance7:        $A_{i,j}^{\left(k\right)}\leftarrow \exp\left(-d_{qk}^{2}/\tau \right)$ // Gaussian weight8:    **end for**9:    $A_{i,j}^{\left(k\right)}\leftarrow A_{i,j}^{\left(k\right)}/\sum_{k}A_{i,j}^{\left(k\right)}$ // Normalize10:   $F_{i,j}^{\left(h\right)}\leftarrow \sum_{k}A_{i,j}^{\left(k\right)}\cdot F_{i,j+k\cdot d_{h}}$ // Weighted aggregation11:**end for**12:// Vertical Attention13:**for** each position (i,j) **do**14:    **for** k=−⌊K/2⌋ to ⌊K/2⌋ **do**15:        $d_{qk}\leftarrow \left|r_{i,j}-r_{i+k\cdot d_{v},j}\right|$ // Range distance16:        $A_{i,j}^{\left(k\right)}\leftarrow \exp\left(-d_{qk}^{2}/\tau \right)$ // Gaussian weight17:    **end for**18:    $A_{i,j}^{\left(k\right)}\leftarrow A_{i,j}^{\left(k\right)}/\sum_{k}A_{i,j}^{\left(k\right)}$ // Normalize19:    $F_{i,j}^{\left(v\right)}\leftarrow \sum_{k}A_{i,j}^{\left(k\right)}\cdot F_{i+k\cdot d_{v},j}^{\left(h\right)}$ // Weighted aggregation20:**end for**21:$F_{out}\leftarrow F^{\left(v\right)}$22:**return**$F_{out}$

### 3.4. Geometry-Context Modulation

An intuitive idea is to directly aggregate features from geometrically similar neighborhoods using geometric cues. However, this hard aggregation strategy is suboptimal—geometric similarity does not necessarily imply semantic similarity. For example, two spatially adjacent points may lie on the same plane yet belong to entirely different object categories; directly fusing their features based on spatial similarity would undermine semantic discriminability and blur object boundaries. To address this, we propose a progressive geometric information incorporation mechanism: instead of letting geometric cues dominate feature aggregation, we employ adaptive gating to enhance or suppress original features—fusing geometric and semantic information to generate spatial modulation maps for adaptive feature recalibration. Specifically, the context feature C obtained after geometry-guided aggregation encodes both geometric structural information (injected by the geometry attention module) and semantic content (from features extracted by the 2D backbone). Using this hybrid context, a lightweight convolutional head ϕ(·) predicts a dense modulation map $M\in R^{B\times C\times H\times W}$:

(11) $$M=\phi \left(C\right)=\sigma \left({\mathrm{Conv}}_{1\times 1}\left(\mathrm{GELU}\left({\mathrm{DWConv}}_{3\times 3}\left(C\right)\right)\right)\right),$$

where ${\mathrm{DWConv}}_{3\times 3}$ denotes the depthwise separable convolution for capturing local spatial patterns, and σ(·) is the sigmoid activation function that constrains the modulation coefficients to the interval (0,1). The modulation map is then applied to the original features F via a residual gating mechanism:

(12) Y=F⊙(1+M),

where ⊙ denotes element-wise multiplication. This design ensures that the identity mapping is preserved when M≈0, while allowing the network to selectively enhance information-rich regions when M>0. By conditioning the modulation map on geometry-aware context—rather than raw geometric distances—GCM achieves a more flexible and semantically consistent feature enhancement, jointly considering geometric structure and semantic coherence.

### 3.5. Model Architecture

As shown in Figure 2c, combining the geometry attention module and the geometry-context modulation module yields the GE Block, a geometry enhancement module that can be embedded into any 2D backbone network. Each module takes a range image with the same spatial dimensions as the input feature map to compute geometric priors. By inserting this enhancement module after the feature extraction blocks of a standard 2D backbone, we obtain the proposed Geometry-Enhanced Network, GENet. Specifically, we design two integration strategies: the lightweight version inserts a GE Block only at the output of each backbone stage for feature calibration; the standard version inserts a GE Block after each feature extraction block for fine-grained geometry-semantic fusion. Figure 2a illustrates the overall architecture of the proposed GENet.

### 3.6. Range Interpolation

When projecting 3D LiDAR point clouds onto 2D range images, the sparsity and non-uniformity of LiDAR sampling inevitably result in numerous holes in the generated range images. To mitigate this issue, we propose a range view interpolation strategy. For each pixel in candidate regions, we query its *k* nearest neighbors from the valid projected point set using KD-Tree, and compute the interpolation via inverse distance weighting (IDW):

(13) $${\hat{x}}_{i}=\sum_{j\in N_{k}\left(i\right)}w_{ij}\cdot x_{j},\;w_{ij}=\frac{1/d_{ij}}{\sum_{j^{\prime }}1/d_{ij^{\prime }}},$$

where $d_{ij}$ denotes the Euclidean distance between pixel *i* and its neighbor *j* on the image plane. This simple yet effective interpolation operation densifies the range image while preserving local geometric consistency, enabling subsequent convolutional layers to operate on more complete and coherent inputs.

## 4. Results

### 4.1. Experimental Setup

We conduct experiments on two popular benchmark datasets. SemanticKITTI [16] is the most widely used large-scale benchmark for point cloud semantic segmentation, built upon the KITTI Odometry dataset, containing 22 sequences with 43,552 frames and 19 semantic classes (including roads, buildings, vehicles, pedestrians, vegetation, and other common objects in urban driving scenes). Sequences 00–10 are used for training, sequence 08 for validation, and sequences 11–21 for testing. SemanticPOSS [17] is a more challenging small-scale dataset with 2988 frames and 14 semantic classes. Its scenes contain more dynamic objects and sparser point cloud distributions, making it suitable for evaluating model generalization.

For implementation details, all experiments are conducted on a single NVIDIA V100 GPU. We use AdamW as the optimizer with a cosine annealing scheduler and a learning rate of 0.001. Weight decay is set to 0.001. The model is trained for 80 epochs on SemanticKITTI and 100 epochs on SemanticPOSS, with a batch size of 8.

### 4.2. Results

**Figure 3:**
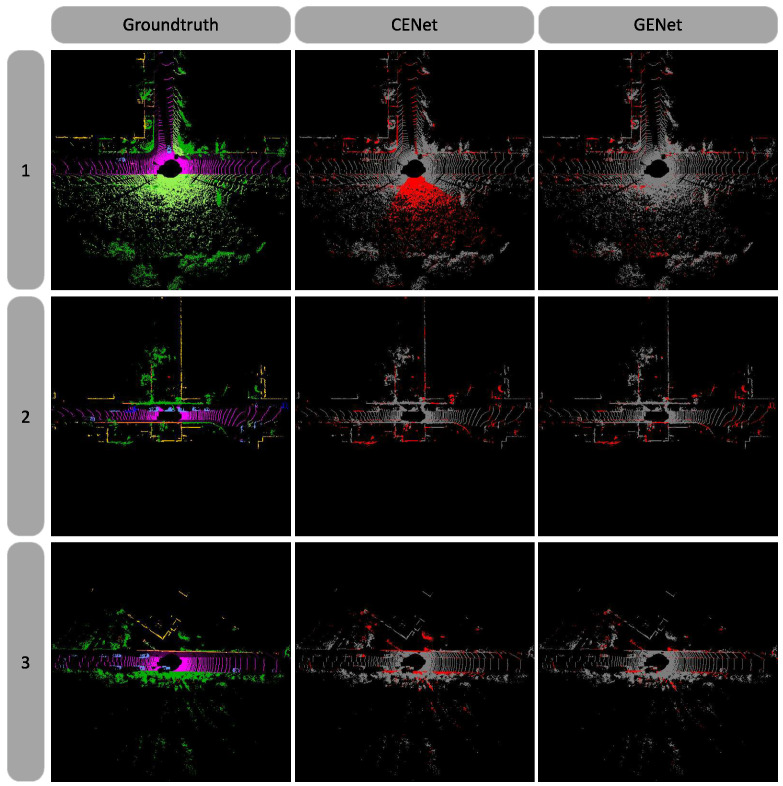
Qualitative analysis on the SemanticKITTI validation set. From left to right, it shows ground truth labels, CENet’s segmentation error map (red indicates incorrect predictions), and our proposed method in the current frame. The segmentation error map is highlighted in red for better visualization of errors. The proposed method achieves more accurate geometric boundary preservation compared to CENet, resulting in a significant improvement in overall segmentation performance.

Experimental results of GENet on the SemanticKITTI benchmark are shown in Table 1, reporting mean IoU, per-class IoU, and FPS. Compared with multi-view methods, although our method does not show significant performance advantages, it clearly outperforms in terms of parameter count (6.9 M) and real-time capability (41.6 FPS). Compared with voxel-based and projection-based methods, GENet improves segmentation accuracy (67.4%) while maintaining real-time performance, achieving an optimal trade-off between efficiency and accuracy. Notably, GENet-Light achieves the fastest inference speed (41.6 FPS) while still outperforming previous best results among range view projection methods. Table 2 presents quantitative comparison results on the SemanticPOSS dataset. Due to its smaller scale and sparser distribution, the performance gaps among methods are relatively small; nevertheless, our method still shows improvement over the baseline on this dataset. Figure 3 presents qualitative comparison with the baseline model CENet, showing that our method is more accurate in preserving geometric boundaries and achieves notable improvement in overall segmentation quality.

### 4.3. Ablation Study

To quantitatively analyze the effectiveness and necessity of each proposed component, we conduct comprehensive ablation studies. Following previous works, experiments are performed on the SemanticKITTI validation set for efficient training and comparison, with 2D resolution fixed at 64 × 512.

We first compare the performance impact of different information enhancement strategies on the 2D backbone of range view methods: concatenating range residual matrices to the input (representing input-level fusion), inserting CBAM blocks at each backbone stage (representing semantically-guided feature fusion), and our proposed method (geometry-guided feature fusion). All these enhancement strategies improve the performance of the baseline model CENet, indicating that existing 2D backbones still cannot fully extract point cloud information. Our method integrates both semantic and geometric cues, among which GENet-Light achieves the fastest inference speed (41.6 FPS), GENet-Standard achieves the highest segmentation accuracy (65.4%) and real-time performance still outperforms other methods with the same accuracy. The results are shown in Table 3.

We further analyze the impact of individual components, including the geometry attention aggregation module, geometry modulation module, and range interpolation algorithm. CENet [8] is adopted as the baseline, and we compare performance metrics under different configurations, including mIoU and FPS. The second row reports the effectiveness of the range interpolation algorithm, which searches for potential interpolation points via morphological operations and performs nearest neighbor label assignment, significantly alleviating the sparsity issue of point clouds. As shown, this method gets 0.5% mIoU improvement. The third row reports results of applying the geometry attention aggregation module alone. Unfortunately, using this module alone for feature aggregation only brings a 0.7% improvement. We attribute this to the fact that geometric similarity does not equate to semantic similarity; thus, forcing feature aggregation based solely on geometric relations cannot achieve optimal results. The fourth row reports results of applying the context modulation module to 2D backbone features alone, yielding approximately 0.3% improvement. This is because when only semantic information is input, the module essentially functions as a lightweight spatial attention module similar to CBAM; however, the texture information in range images contains substantial noise, making it difficult to achieve optimal results using semantic information alone. When the geometry attention aggregation module and context modulation module are applied together, a 2.2% mIoU improvement is achieved, demonstrating the effectiveness of our proposed progressive strategy for fusing semantic and geometric information. Component ablation results are shown in Table 4.

Finally, we discuss the generalization of the proposed module across different backbones. We select the modern convolutional network ConvNeXt V2 [29] as the experimental backbone. Compared with ResNet, ConvNeXt V2 incorporates successful design principles from Vision Transformer, comprehensively modernizing the pure convolutional architecture and achieving stronger representation capability, making it a strong baseline for various vision tasks. Experiments with ConvNeXt V2-atto achieve 62.9% mIoU, outperforming the CENet+HardNet baseline with comparable parameter counts. This demonstrates the generalization of our proposed module across different backbones, while also establishing a new lightweight baseline. Results are shown in Table 5.

## 5. Conclusions

This work proposes a Geometry-Enhanced Network called GENet for LiDAR point cloud segmentation, the core components of which can be integrated into any 2D backbone. The method contains two core components: the Geometry Attention Module uses range information to explicitly model geometric relationships among points and guides semantic feature aggregation accordingly; the Geometry-Context Modulation (GCM) module uses an adaptive modulation mechanism to fuse geometric information while preserving the semantic representation ability of the backbone. We also propose a new range view interpolation strategy to solve the hole problem when projecting point clouds to 2D images. Experiments show that our method achieves a good balance between efficiency and performance.

## Figures and Tables

**Table 1:** The performance comparison on SemanticKITTI test set.

Category	Methods	FPS (Hz)	Mean-IoU	Car	Bicycle	Motorcycle	Truck	Other-Vehicle	Person	Bicyclist	Motorcyclist	Road	Parking	Sidewalk	Other-Ground	Building	Fence	Vegetation	Trunk	Terrain	Pole	Traffic-Sign
Point-based	PointNet++ [1]	0.1	20.1	53.7	1.9	0.2	0.9	0.2	0.9	1.0	0.0	72.0	18.7	41.8	5.6	62.3	16.9	46.5	13.8	30.0	6.0	8.9
	RandLa-Net [4]	20	53.9	94.2	26.0	25.8	40.1	38.9	49.2	48.2	7.2	90.7	60.3	73.7	20.4	86.9	56.3	81.4	61.3	66.8	49.2	47.7
	KPConv [3]	−	58.8	96.0	32.0	42.5	33.4	44.3	61.5	61.6	11.8	88.8	61.3	72.7	31.6	95.0	64.2	84.8	69.2	69.1	56.4	47.4
	BAAF [18]	5	59.9	95.4	31.8	35.5	48.7	46.7	49.5	55.7	53.0	90.9	62.2	74.4	23.6	89.8	60.8	82.7	63.4	67.9	53.7	52.0
Voxel-based	SPVNAS [19]	8.9	66.4	97.3	51.5	50.8	59.8	58.8	65.7	65.2	43.7	90.2	67.6	75.2	16.9	91.3	65.9	86.1	73.4	71.0	64.2	66.9
	Cylinder3D [20]	6.2	68.9	97.1	67.6	63.8	50.8	58.5	73.7	69.2	48.0	92.2	65.0	77.0	32.3	90.7	66.5	85.6	72.5	69.8	62.4	66.2
Multi-view	RPVNet [12]	7.9	70.3	97.6	68.4	68.7	44.2	61.1	75.9	74.4	73.4	93.4	70.3	80.7	33.3	93.5	72.1	86.5	75.1	71.7	64.8	61.4
	2DPASS [13]	8.4	72.9	97.0	63.6	63.4	61.1	61.5	77.9	81.3	74.1	89.7	67.4	74.7	40.0	93.5	72.9	86.2	73.9	71.0	65.0	70.4
Image-based	SqueezeSeg-CRF [21]	55	30.8	68.3	18.1	5.1	4.1	4.8	16.5	17.3	1.2	84.9	28.4	54.7	4.6	61.5	29.2	59.6	25.5	54.7	11.2	36.3
	SqueezeSegV2-CRF [22]	40	39.6	82.7	21.0	22.6	14.5	15.9	20.2	24.3	2.9	88.5	42.4	65.5	18.7	73.8	41.0	68.5	36.9	58.9	12.9	41.0
	RangeNet++ [6]	12.8	52.2	91.4	25.7	34.4	25.7	23.0	38.3	38.8	4.8	91.8	65.0	75.2	27.8	87.4	58.6	80.5	55.1	64.6	47.9	55.9
	MPF [23]	20.6	55.5	93.4	30.2	38.3	26.1	28.5	48.1	46.1	18.1	90.6	62.3	74.5	30.6	88.5	59.7	83.5	59.7	69.2	49.7	58.1
	SqueezeSegV3 [14]	6	55.9	92.5	38.7	36.5	29.6	33.0	45.6	46.2	20.1	91.7	63.4	74.8	26.4	89.0	59.4	82.0	58.7	65.4	49.6	58.9
	FIDNet [7]	33.7	58.6	93.0	45.7	42.0	27.9	32.6	62.6	58.1	30.5	90.8	58.3	74.9	20.1	88.5	59.5	83.1	64.3	67.8	52.6	60.0
	SalsaNext [9]	24	59.5	91.9	48.3	38.6	38.9	31.9	60.2	59.0	19.4	91.7	63.7	75.8	29.1	90.2	64.2	81.8	63.6	66.5	54.3	62.1
	KPRNet [24]	0.3	63.1	95.5	54.1	47.9	23.6	42.6	65.9	65.0	16.5	93.2	73.9	80.6	30.2	91.7	68.4	85.7	69.8	71.2	58.7	64.1
	Lite-HDSeg [25]	20	63.8	92.3	40.0	55.4	37.7	39.6	59.2	71.6	54.3	93.0	68.2	78.3	29.3	91.5	65.0	78.2	65.8	65.1	59.5	67.7
	RangeViT [26]	10	64.0	95.4	55.8	43.5	29.8	42.1	63.9	58.2	38.1	93.1	70.2	80.0	32.5	92.0	69.0	85.3	70.6	71.2	60.8	64.7
	CENet [8]	37.8	64.7	91.9	58.6	50.3	40.6	42.3	68.9	65.9	43.5	90.3	60.9	75.1	31.5	91.0	66.2	84.5	69.7	70.0	61.5	67.6
	**GENet-Light**	**41.6**	**66.3**	96.7	57.3	59.4	48.8	57.5	74.7	79.6	14.9	90.4	61.8	74.8	29.5	90.7	66.0	85.8	72.7	70.0	60.6	67.9
	**GENet**	**24.8**	**67.4**	96.5	62.3	52.3	47.4	48.8	72.4	76.1	39.0	90.3	60.0	76.1	35.2	93.1	70.4	85.7	72.8	70.6	63.9	67.8

**Table 2:** The performance comparison on SemanticPOSS test set.

	Person	Rider	Car	Truck	Plants	Traffic Sign	Pole	Trashcan	Building	Cone/Stone	Fence	Bike	Ground	mIoU
SqueezeSeg [21]	14.2	1.0	13.2	10.4	28.0	5.1	5.7	2.3	43.6	0.2	15.6	31.0	75.0	18.9
SqueezeSegV2 [22]	48.0	9.4	48.5	11.3	50.1	6.7	6.2	14.8	60.4	5.2	22.1	36.1	71.3	30.0
RangeNet53 [6]	57.3	4.6	35.0	14.1	58.3	3.9	6.9	24.1	66.1	6.6	23.4	28.6	73.5	30.9
MINet [27]	62.4	12.1	63.8	22.3	68.6	16.7	30.1	28.9	75.1	28.6	32.2	44.9	76.3	43.2
FIDNet [7]	72.2	23.1	72.7	23.0	68.0	22.2	28.6	16.3	73.1	34.0	40.9	50.3	79.1	46.4
CENet [8]	75.5	22.0	77.6	25.3	72.2	18.2	31.5	48.1	76.3	27.7	47.7	51.4	80.3	50.3
Cylinder3D [20]	75.9	30.0	75.8	28.7	75.7	29.5	37.2	36.7	82.3	34.1	47.5	53.9	80.1	52.9
MinkNet [28]	77.8	29.1	81.3	33.9	75.2	22.0	42.5	36.4	80.7	23.9	51.2	57.5	79.1	53.1
**GENet-Light**	74.1	24.3	78.6	27.6	71.7	23.2	33.6	31.0	79.4	37.6	46.3	52.8	77.5	**50.6**
**GENet**	75.2	26.1	79.0	29.0	73.4	25.4	34.1	32.5	80.0	39.8	47.1	53.6	78.6	**51.8**

**Table 3:** Performance comparison of different information enhancement methods.

Enhancement Method	mIoU (%)	FPS
Baseline	62.0	52.3
+ Concat	63.3	51.5
+ CBAM	63.6	23.8
+ GENet-Light	64.2	41.6
+ GENet-Standard	**65.4**	24.7

**Table 4:** Ablation study results on SemanticKITTI validation set.

	RI ^†^	ASRA	GCM	mIoU (%)	FPS
Baseline	×			62.0	52.3
Ours	✓			62.5	51.8
	✓	✓		62.7	43.2
	✓		✓	62.3	48.5
	✓	✓	✓	**64.2**	41.6

^†^ RI: Range Interpolation. × indicates using the original interpolation from CENet; ✓ indicates using our proposed IDW interpolation.

**Table 5:** Performance comparison of different backbones.

Backbone	mIoU (%)	Params (M)
CENet + HardNet	60.0	6.8
CENet + ConvNextV2-atto	61.2	4.2
GENet-Light + ConvNextV2-atto	**62.9**	4.5

## Data Availability

The datasets analyzed in this study are publicly available. SemanticKITTI dataset is available at http://www.semantic-kitti.org/ (accessed on 10 February 2026) and SemanticPOSS dataset is available at http://www.poss.pku.edu.cn/download.html (accessed on 10 February 2026).
